# Bridging Formal Shape Models and Deep Learning: A Novel Fusion for Understanding 3D Objects

**DOI:** 10.3390/s24123874

**Published:** 2024-06-15

**Authors:** Jincheng Zhang, Andrew R. Willis

**Affiliations:** Department of Electrical and Computer Engineering, University of North Carolina at Charlotte, Charlotte, NC 28223, USA; arwillis@charlotte.edu

**Keywords:** shape grammars, deep learning, procedural modeling, parametric modeling, synthetic data

## Abstract

This article describes a novel fusion of a generative formal model for three-dimensional (3D) shapes with deep learning (DL) methods to understand the geometric structure of 3D objects and the relationships between their components, given a collection of unorganized point cloud measurements. Formal 3D shape models are implemented as shape grammar programs written in Procedural Shape Modeling Language (PSML). Users write PSML programs to describe complex objects, and DL networks estimate the configured free parameters of the program to generate 3D shapes. Users write PSML programs to enforce fundamental rules that define an object class and encode object attributes, including shapes, components, size, position, etc., into a parametric representation of objects. This fusion of the generative model with DL offers artificial intelligence (AI) models an opportunity to better understand the geometric organization of objects in terms of their components and their relationships to other objects. This approach allows human-in-the-loop control over DL estimates by specifying lists of candidate objects, the shape variations that each object can exhibit, and the level of detail or, equivalently, dimension of the latent representation of the shape. The results demonstrate the advantages of the proposed method over competing approaches.

## 1. Introduction

The generation and understanding of three-dimensional (3D) geometries hold significant importance across diverse domains, from computer graphics to robotics and virtual reality. Recent advancements in deep learning (DL) and generative modeling have propelled research in the area of 3D shape generation. Notably, techniques such as Variational Autoencoders (VAEs) [1,2,3], 3D Generative Adversarial Networks (3D-GANs) [4,5,6], and 3D Stable Diffusion [7,8,9,10] have shown promise in autonomously producing realistic and diverse 3D shapes. Shape grammars have also been demonstrated as a powerful approach for formal model generation by providing a rule-based framework for generating complex geometric structures and enforcing constraints within objects [11,12,13]. Each methodology has its advantages and limitations. This article seeks to provide a fusion of these two methodologies to achieve the best of both worlds for novel 3D shape synthesis.

**Figure 1:**
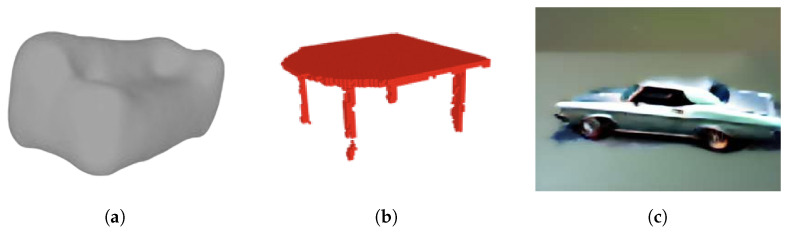
Deep learning methods face significant challenges in grasping the geometric and physical constraints inherent in 3D objects, resulting in shortcomings in the generated objects. (**a**) A VAE generated couch model only provides a rough approximation of the complex geometry of a couch [1]. (**b**) A 3D GANs generated table model lacks manifold geometry for the legs and fails to enforce self-similarity constraints, resulting in variations in shape and size among the four legs [4]. (**c**) A stable diffusion generated car model is smoothed on the object edges and fails to adhere to real-world constraints, as one of the front wheels occupies the spatial location intended for the car’s front fender [9].

Despite the progress achieved, challenges and limitations persist in the deep learning 3D shape generation methodology. Figure 1 showcases several failure instances of these methodologies. VAEs, as evidenced in Figure 1a, often struggle with capturing complex and high-dimensional distributions of 3D shapes. GANs often struggle with mode collapse in training, where the generator produces a limited diversity of shapes or collapses to a few modes, failing to capture the full distribution of the data as shown in Figure 1b. Mode collapse can make GANs difficult to train and lead to the generation of unrealistic or repetitive shapes, limiting the variety and quality of the generated outputs. Figure 1c shows a car model generated by 3D stable fusion techniques which may struggle with preserving fine-grained details and local geometric features, leading to information loss in complex shapes or smoothed shapes.

Shape grammars have been used commonly in computer graphics and design to describe the generation of complex shapes through a set of production rules [14,15]. These rules define how basic shapes or components can be combined and manipulated to form more intricate structures. Shape grammars provide a systematic approach to generating shapes by specifying the relationships between various components and enforcing constraints to ensure the coherence and consistency of the generated designs.

Shape grammar rules can be implemented using the modeling language as the formal syntax and vocabulary [16,17,18,19]. Users can define programs that describe objects as a semantic hierarchy of 3D shape elements, where each element may be a semantic group of objects, e.g., a floor of a building, or an indivisible object, i.e., a brick within a building. Each indivisible object, e.g., a brick from a building, is modeled in terms of its geometry and appearance.

Shape grammars offer a means to encode the implicit generation rules and geometric constraints inherent in objects, which remains challenging for deep learning models to grasp. However, common objects typically have predictable generation rules which are satisfied by all instances of these objects. For example, tables usually feature a top surface and multiple supporting legs connected to the top, and cars typically have four wheels on two sides that can roll. However, deep learning neural networks find it challenging to comprehend these constraints, hindering their ability to accurately generate such objects. Shape grammars provide a solution by implementing these rules to construct objects and allowing users to define the parameters of these rules.

Bridging deep learning techniques with shape grammars presents significant potential. Users can define shape programs and convey shape rules to artificial intelligence (AI) systems. By employing DL networks to learn the parameters of these rules rather than the rules themselves, AI models gain the ability to internalize such constraints. This capability leads to disentangled representations within the latent space, where each dimension corresponds to a meaningful attribute of the data. This promises enhanced controllability and interpretability of the generated shapes.

This article proposes a novel fusion of 3D shape representation using shape grammars and DL model estimation. Shapes are represented as a formal shape grammar using Procedural Shape Modeling Language (PSML) [19], which applies a sequence of rules to construct a 3D geometric model as a collection of 3D primitives. In contrast to competing approaches from the DL literature, the inclusion of dynamic parameterized formal shape models promises to allow DL applications to more accurately represent the structure of commonplace objects.

The proposed method merges the powerful representation of PSML for many important shape-understanding contexts with the heretofore unprecedented capabilities for non-linear estimation provided by recent advances in AI through deep learning neural models and their training methodologies. Prior efforts have seen much success in deterministically applying shape grammars in procedural modeling contexts [11,20] to generate impressive geometric models with direct human input and control. However, the capability to leverage procedural shape grammar models to solve the inverse problem of the shape estimation approach from measured data has eluded academics in terms of being able to extract reliable fits of shape grammar parameters given measured depth, RGB, LiDAR, or other data [21,22,23]. The promise of using the unprecedented power of AI estimation to solve this inverse problem has not been investigated. This article explores the extent to which current DL models are capable of solving the problem of estimating shape grammar parameters from measured data. Our results demonstrate that the deep learning models effectively address this new problem, achieving reliable and accurate parameter estimation.

In this article, we demonstrate several benefits of our approach that fuses shape models with DL estimation which are listed below:

- Shape estimates are guaranteed to satisfy complex geometric shape and physical constraints, including self-symmetry, self-similarity, and free-standing stability properties.
- Shape estimates are guaranteed to satisfy important geometric model properties by providing water-tight, i.e., manifold, polygon models that require a small number of triangle primitives to describe the basic object geometry.
- Shape estimates provide a highly compact parametric representation of objects, allowing objects to be efficiently shared over communication links.
- User-provided shape programs allow human-in-the-loop control over DL estimates. Aspects of this control include specifying lists of candidate objects, the shape variations that each object can exhibit, and the level of detail or, equivalently, dimension of the latent representation of the shape. These aspects of our approach allow humans to more easily control the DL estimate outputs and also enable humans to more easily interpret DL estimate results, which we collectively refer to as “human-in-the-loop” benefits.
- Users can control the complexity and diversity of DL-estimated shapes for each object and for each object component directly through the construction of the DL network.
- Object models can be used to synthesize training data for DL systems, improving over current 3D model databases which use static 3D models and therefore lack geometric diversity. Object models can be combined to generate extremely large synthetic 2D/3D datasets having rich geometric diversity and including important annotations to support a wide variety of 3D and 2D DL applications.
- An example of the proposed DL fusion is provided that detects objects, and their parametric representation given a PSML shape grammar is demonstrated. Key metrics for the model estimates are shown that demonstrate the benefits of this approach.

These contributions open the door to the integration of shape-grammar-based data generation methods with deep learning techniques for 3D object/scene understanding. User-defined shape programs offer various benefits and advantages over competing approaches which are demonstrated by the results of this study.

## 2. Related Work

This study explores the advantages of shape grammar in data synthesis compared to other methods of data generation. Through experiments, the PSML-driven data generation approach shows significant potential for various computer vision applications. For these reasons, a review of the literature related to this article is divided into two parts:

- A overview of shape grammar and its applications.
- An examination of deep learning generative models of 3D shapes.

### 2.1. Shape Grammar

Shape grammar, proposed in the 1970s [24], provides a formal framework for generating and analyzing complex shapes and designs. As as a shape-based visual description grammar and a rule-based automated design grammar, shape grammar has been applied to many domains, including urban planning [12,13], industrial design [14], and computer-aided design [15]. Over the years, advancements in shape grammar have led to the development of sophisticated methods for shape generation [25], analysis [26], and optimization [27], integrating computational techniques such as procedural modeling [19], parametric design [28], and machine learning [29].

There has been a considerable amount of work that investigates the use of shape grammars for vision tasks with a large number of articles being produced that focus on the segmentation of architecture within images [30,31] or segmentation of building facade images [21,22,23,32]. However, these techniques were limited to 2D grammars since the labeled primitives produced by the used grammars were limited to 2D faces. Other work leverages shape grammar to model 3D indoor scenes from point clouds [33]. Recently, researchers have been leveraging shape grammar to guide 3D shape semantic labeling [34] or scene graph generation [35]. This study systematically explores the benefits of the data generation method driven by the shape grammar and its potential in deep learning computer vision tasks and machine understanding, where an AI system emulates the sense-making and decision-making ability of human beings.

### 2.2. Generative Models of 3D Shapes

Deep learning generative models have significantly advanced the field of 3D shape generation, with methodologies such as Variational Autoencoders (VAEs) [1,2,3], Generative Adversarial Networks (GANs) [4,5,6], and Stable Diffusion [7,8,9,10] emerging as popular methodologies. VAEs encode input shapes into a latent space and reconstruct them via a decoder but often struggle with capturing complex and high-dimensional distributions of 3D shapes. GANs leverage a generator–discriminator framework to produce realistic shapes but may suffer from mode collapse leading to the generation of unrealistic or repetitive shapes. Stable diffusion, a recent innovation that offers improved training stability and control over generated samples, may struggle with capturing fine-grained details and preserving the complex geometric properties present in real-world objects.

These methods train the AI systems to learn the geometric constraints such as self-similarity, and physical constraints such as the free-standing stability of the 3D objects. This is extremely difficult for AI systems, as their training data typically do not encode these rules or principles. It results in these methods tending to generate an approximation of instances of objects but not a geometric model that adheres to real-world constraints. The fundamental difference of this study is to encode into training data the organizations of objects in terms of their components and their relationships to other objects.

## 3. Methodology

The methodology of this article is organized into the following sections:

- An introduction to the Procedural Shape Modeling Language (PSML) that incorporates shape grammar programs as elements within the sequential programming code (Section 3.1).
- A discussion of the benefits offered by the PSML data generation approach (Section 3.2).
- A fused system of PSML and DL that takes point cloud as input and generates 3D shape estimates of objects (Section 3.3).
- An application of using the PSML-driven method to generate synthetic datasets (Section 3.4).

### 3.1. Procedural Shape Modeling Language (PSML)

PSML [19] is a programming language to generate 3D shapes procedurally based on shape grammar rules. It provides programmers the ability to describe shapes in terms of their 3D elements, where each element may be a semantic group of 3D objects, e.g., a brick wall, or an individual object, e.g., an individual brick. As such, users may query these models for volumetric information such as the number, position, orientation, and volume of 3D elements. PSML grammar associates labels to both object space, i.e., geometric objects, and void space, i.e., the space that bounds objects. These labels may be used to facilitate analysis that requires knowledge of both types of information, e.g., furniture placement, accessibility, navigation of virtual spaces, and path planning.

Algorithm 1 shows an example of a PSML program to generate a table object. The overall structure of a PSML program includes one “ShapeGrammar” declaration that contains one or more “method” declarations. Each method declaration must include at least one “rules” declaration. Rule blocks must be defined within each method, and each rules block contains a set of shape grammar production rules. The execution of a production rule causes the non-terminal symbol referred to as the predecessor to be replaced by the successor, which may be one or more terminal or non-terminal symbols. Terminal symbols, indicated by the special string “terminal” in PSML algorithms, do not appear as predecessors in any production rule and are visible elements that exist in the final 3D model except terminals declared to be space. PSML is constrained to work entirely from closed geometries and associates semantic labels to non-terminals, visible terminals, and empty spaces. In PSML, terminals are drawn from a pre-defined set of 3D shape primitives, for example, box, cylinder, sphere, and cone, and non-terminals are constructed from multiple terminals to represent complicated shapes. Shape grammars specified within the rule blocks use the passed shape, argument variables, and locally defined variables to generate an instance of the grammar shape.
**Algorithm 1:** The contents of the PSML program: Table.psm
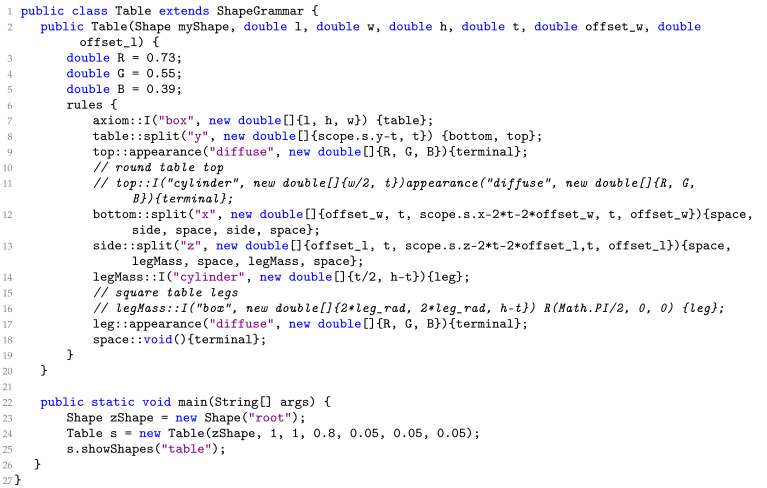


**Figure 2:**
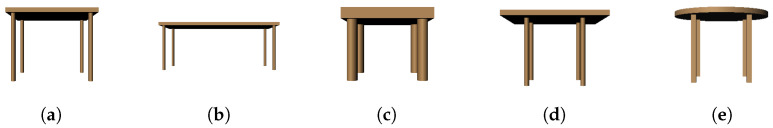
Semantic variations of the table models generated by different PSML program parameters. (**a**) A visualization of the shape generated by Table.psm (Algorithm 1). (**b**) A variation of l=2. (**c**) A variation of t=0.12. (**d**) A variation of offset_l=offset_w=0.12. (**e**) A variation with round top and square legs.

Algorithm 1 generates a “table” shown in Figure 2a. With the rule block of the table method, a box is first defined (line 7). The box is then split into two sections “top” (line 9) and “bottom” (line 12) with the “top” being the surface of the table and the bottom the space where the table legs are. The algorithm then creates a space in the center of the “bottom” part and only keeps two faces on the side of the plane where the legs are (line 13). These two sides then respectively get cut in the middle to finally create two table legs on each side (line 14). The appearance of the table is colored brown (lines 9 and 17). The Table.psm generates a table object with a square top and four round legs. The commented lines (lines 10, 11, 15, and 16) offer the opportunity to generate a table object with different semantic structures, a round top, and square legs. In this example, the tabletop (line 19) and legs (line 17) are the “terminals” of the program and all other symbols are non-terminal symbols, which are replaced by terminal or non-terminal symbols.

Figure 2 shows various realizations of the table object and demonstrates how the representation guarantees that target shapes satisfy the shape constraints over all possible parameter variations, e.g., there are always table legs connecting to a surface for these variations. More generally, the PSML syntax for detecting the size and position of the current volume allows the user to develop shapes that re-organize their components consistently over parametric variations, e.g., anisotropic scaling.

### 3.2. Benefits of PSML-Driven Data Generation

Shapes represented using PSML offer significant benefits, including the following:

- Enforced geometric constraints and physical constraints;
- Manifold polygon models;
- Compact parametric representation;
- Unlimited semantic variability;
- Human-in-the-loop control and interpretability of DL estimates.

Each of these benefits will be discussed in the following subsections.

#### 3.2.1. Geometric and Physical Constraints

Shapes generated using PSML are guaranteed to adhere to the geometric and physical constraints, including self-symmetry, self-similarity, and free-standing stability. By incorporating these constraints into the generation process, PSML ensures that the resultant shapes not only exhibit desired geometric properties but also possess structural integrity and functional coherence.

In the provided table example, the four legs are generated from the same shape primitive “cylinder” and the same parameters *t* and *h* (Algorithm 1 line 14). By employing this generation approach, PSML ensures that all legs exhibit precisely the same shape, thereby guaranteeing uniformity among them. This geometric constraint mirrors real-world manufacturing practices commonly employed in producing table objects, where consistency in leg design is important for structural stability.

PSML programming also allows components of objects to be constructed with appropriate relative positions. The relative position of the legs is delineated by the common parameters, denoted as offset_l and offset_w (Algorithm 1 line 13). These geometric parameters define the spatial arrangement of the legs, ensuring consistency and symmetry in their positioning relative to each other and to the table top they support. The precise relative positions of the legs, together with the uniform shapes, promote balanced weight distribution and stability of the table, ensuring its suitability for applications such as real-world simulation.

**Figure 3:**
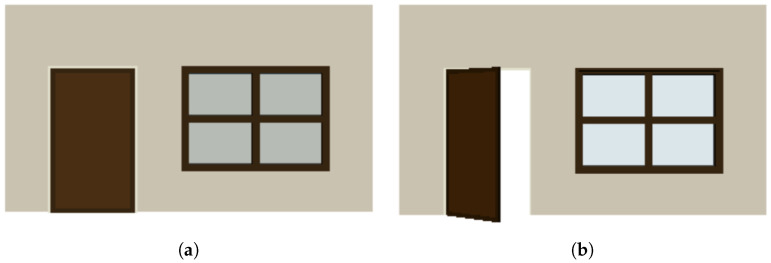
Shape grammar representation allows for the systematic generation of doors with realistic interactive behavior. (**a**) A closed door. (**b**) An open door.

The constraints not only apply to individual objects but can also extend across different objects. In Figure 3, an example illustrates how a door generated using PSML can open and close within a wall. The PSML program for generating the door is outlined in Algorithm 2. The grammar rules (lines 2–5) dictate the mechanism where the door can open or close by rotating along its side connected to the wall. By incorporating these constraints, the PSML program enables doors to exhibit realistic behavior when interacting with other objects, ensuring that the generated doors adhere to principles of real-world physics.
**Algorithm 2:** The contents of PSML program: Door.psm
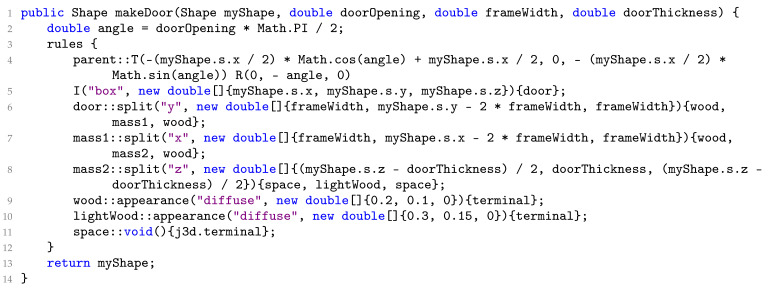


The proposed shape-grammar-driven approach aims to leverage DL networks to estimate the parameters of the predefined rules and constraints that formal grammar generative models rely on to generate shapes. These rules with parameters dictate how basic shapes or components can be combined and transformed to create more complex structures. Deep learning generative models, including 3DGANs, 3DSVAE, and SJC, leverage neural networks to learn patterns and distributions from data. They can generate new content by learning from large datasets and then generating samples that mimic the statistics of the training data, while often struggling to ensure the generation of 3D models that adhere to essential geometric constraints as shown in Figure 1.

#### 3.2.2. Manifold Polygon Models

Shapes generated by PSML are water-tight, i.e., manifold, models. This is attributed to PSML-generating objects from volumetric geometries, characterized by a pre-defined set of 3D closed-shape primitives such as boxes, cylinders, spheres, and cones. These primitives accurately describe the fundamental geometry of the object. This generation approach provides the ability to generate manifold geometries and represent objects as a semantic hierarchy of 3D shape elements. Other shape representations like point clouds and voxel meshes lack such properties. The components of these representations—points or voxels—operate independently, without constraints to enforce connectivity or the creation of a manifold geometry.

**Figure 4:**
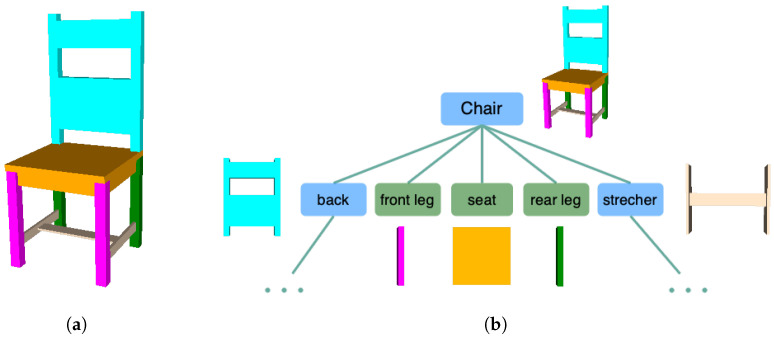
(**a**) An example of PSML constructing a chair hierarchically from its components. (**b**) Derivation tree of the chair. The components of the chair are colored. Shapes represented using PSML are constructed from their components and satisfy the relative constraints of the components.

Figure 4 illustrates a chair instance generated from its hierarchical components. The chair is constructed from a set of components including a seat, front legs, rear legs, a back, and stretchers. The shape derivation tree depicted in Figure 4b showcases the hierarchical composition. The blue nodes represent “non-terminal” objects whose child objects can further refine the shape of their parent objects by substituting the parent shape with one or more terminal or non-terminal shapes. The green nodes represent “terminal” objects, indicating that no further decomposition of this shape is available. These terminal objects, represented by boxes that are closed primitives, construct a chair object with a manifold polygon model. For simplicity, the shape derivation for the back and stretchers is omitted from the derivation tree.

This hierarchical representation of objects allows associating semantic labels to the components, which offers the opportunity for AI systems to understand objects at multiple levels of abstraction, from individual parts to complex assemblies.

#### 3.2.3. Compact Parametric Representation

With pre-defined generation rules, shapes generated using PSML can be represented by a set of parameters that succinctly describe the geometry and appearance of objects. Parametric representations result in the highly compact encoding of object information, minimizing the amount of data that need to be transmitted or stored. This significantly reduces the dimensionality of the data compared to voxel-based or polygonal mesh representations.

The parametric representation provided by PSML offers a lightweight yet powerful solution for encoding object information in resource-constrained environments, making it well suited for applications like mobile robotics, where efficient communication and collaboration are essential. In mobile robotic systems, where the communication bandwidth is often limited, sharing detailed object representations like point clouds or meshes may be impractical due to their high data volume. However, if all robots share knowledge of the grammar of objects or scenes, they can simply communicate the semantic labels and associated parameters to convey their understanding of the scene effectively. By transmitting only the semantic labels and relevant parameters, robots can share information about the scene efficiently while minimizing the data transmission overhead.

#### 3.2.4. Unlimited Semantic Variability

Objects generated using PSML adhere to specific design rules and are defined by a set of parameters. Modifying the shape, size, or appearance of an object can be achieved by simply adjusting these parameters, rather than manipulating complex geometric data directly. This promises the unlimited semantic variability of object models, which can be used to synthesize training data for DL systems, improving current 3D model databases which use static 3D models and therefore lack geometric diversity.

**Figure 5:**
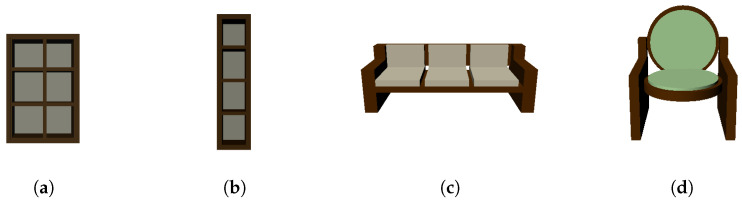
(**a**) A shelf with 3 rows and 2 columns. (**b**) A single-column shelf. (**c**) A regular couch with 3 seats. (**d**) A couch with a round seat and back.

Figure 2 shows different variations of the table object generated by Algorithm 1 using different PSML parameter values (Figure 2b–e) and rules (Figure 2e). The structure of the table object is controlled by the length *l*, the width *w*, the height *h*, the thickness of the tabletop *t*, and the position of the legs, which are determined by the offset from the edges of the table, represented by offset_w and offset_l, respectively. Figure 2a–d show the table variations generated by setting different values to these parameters. Figure 2e visualizes a table variation with a round tabletop and square legs, which are opposite from other tables in Figure 2. More examples of other objects are shown in Figure 5, where variations of shelves and couches are generated by editing associated PSML programs, demonstrating the capability of generating unlimited semantic variations for DL systems. By applying different construction rules and/or passing different parameter values to the generation program of objects, indefinite variations of the object can be synthesized as training data for DL systems.

#### 3.2.5. Human-In-The-Loop Control and Interpretability

User-provided shape programs allow human-in-the-loop control over DL estimates. Aspects of this control include specifying lists of candidate objects, the shape variations that each object can exhibit, and the level of detail or, equivalently, dimension of the latent representation of the shape. Figure 2d–e demonstrate the semantic control by specifying “square table” and “round table” as distinct table realizations. Human-specified shape grammars therefore directly control the label set and implicitly define the shape realizations that can be drawn from the latent representation internal to the trained neural network. Another aspect of Human-In-The-Loop Control is to control the level of detail or, equivalently, the dimension of the latent representation of the shape. In this example, both objects are embedded in a latent feature space having 6 dimensions. Specifically, a total of 6 PSML parameters are used to define both objects including *l*, *w*, *h*, *t*, offset_w, and offset_l. By changing the dimension of the shape grammar parameter vector for shape representation, more simplistic (lower-dimensional) and/or more complex shapes (higher-dimensional) can be specified by humans through shape grammar programs and estimated via neural network training and inference. These controllable aspects of our approach allow humans to more easily control the DL estimate outputs and also enable humans to more easily interpret the DL estimate results, which we collectively refer to as “human-in-the-loop” benefits. Users can control the complexity and diversity of DL-estimated shapes for each object and its components directly through the construction of the DL network.

Through parameterization, shapes generated using PSML allow the formalization of the DL estimate results into specific rules and constraints. Shape grammar rules incorporate parameters that define the properties of the generated elements, such as shape, size, orientation, and position. Parameters can also function within shape grammar rules to enforce constraints during generation. These parameters encode domain-specific knowledge of the shapes, for example, symmetry, alignment, and spatial relationships between components. When discrepancies arise between DL-estimated parameter values and ground truth, these errors serve as indicators of specific knowledge gaps within the model, such as its inability to learn rotational or positional relationships accurately. Analyzing the nature and frequency of these errors allows us to inform model improvement efforts, such as refining the network architecture or augmenting the training data. The transparent attribution of errors to specific aspects of the input data enhances the interpretability of the model’s results, enabling humans to understand and assess its predictions more effectively. This interpretability addresses a crucial challenge in making deep learning systems more comprehensible and trustworthy for real-world applications.

### 3.3. Fusion of PSML and Deep Learning

**Figure 6:**
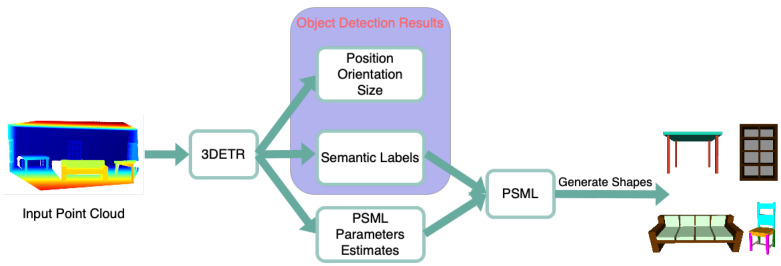
The fused system of PSML and DL for estimating 3D shapes.

Figure 6 illustrates the fused system of PSML and DL for estimating 3D shapes. The proposed system takes the point cloud as input and outputs 3D shape estimates of objects. A DL network, 3DETR [36], is adapted and modified to perform 3D object detection and estimation of the PSML parameters. The estimated parameters, together with the semantic labels for determining the associated shape programs, are passed to the PSML to generate estimates of 3D shapes.

The 3DETR network [36] is an end-to-end Transformer-based object detection model for 3D point clouds. Unlike traditional convolutional neural networks (CNNs) which rely on spatial hierarchies to extract features from images, the 3DETR network leverages the self-attention mechanism of Transformers to capture both spatial and contextual information in an integrated manner. This enables the network to effectively process point cloud data, which lacks the grid-like structure present in images, while also facilitating global context understanding and the precise localization of objects within a 3D scene. The 3DETR network adapts an encoder–decoder architecture that produces a set of features. These features are fed into prediction Multi-Layer Perceptrons (MLPs) to predict bounding boxes. A 3D bounding box contains attributes, including (a) location, (b) size, (c) orientation, and (d) the semantic class of the object.

Two modifications are implemented in the 3DETR network to facilitate the estimation of PSML parameters:

- A new Multi-Layer Perceptron (MLP) is added to the existing architecture for PSML parameters estimation.
- The PSML parameters are encoded as an additional attribute of the 3D bounding boxes for prediction.

In this article, unless specified otherwise, the modified 3DETR network is denoted as 3DETR-P, where P stands for PSML, while 3DETR refers to the original network. The 3DTER-P takes a set of 3D points (point cloud) as input and outputs a set of 3D bounding boxes and associated PSML parameters. A vector of dimension 5 is chosen to encode the PSML parameters representing each object in the dataset. The dimensionality of this vector corresponds to the latent representation of the shape. By adjusting this size, DL networks are enforced to capture finer details of the object when increased, while reducing it provides more flexibility in the estimated solution space.

The $L_{1}$ regression loss, i.e., the mean absolute error (MAE), is used as the loss function to measure the difference between the predicted values and the ground truth values. The equation for the PSML parameter loss is as follows:

(1) $$L_{\mathrm{PSML}}=\frac{1}{n}\sum_{i=1}^{n}\left|p_{i}-{\hat{p}}_{i}\right|$$

where *n* is the number of PSML parameters, $p_{i}$ is the ground truth value for the *i*-th paramter, and ${\hat{p}}_{i}$ is the network predicted value for the *i*-th parameter. This loss was added to the naive 3DETR loss with weight as the new final loss function to train the network. The final loss function is as follows:

(2) $$L=L_{3\mathrm{DETR}}+\lambda L_{\mathrm{PSML}}$$

where λ is the weight associated to the PSML loss, and $L_{3\mathrm{DETR}}$ is defined in [36].

### 3.4. Data Synthesis for DL Systems

For the training of the 3DETR-P deep learning network and the evaluation of the integrated system shown in Figure 6, point cloud data annotated with ground truth information are essential. These include 3D bounding boxes, semantic labels, and shape grammar parameters. However, to our knowledge, there is no publicly available dataset that meets these requirements. It is necessary to generate a new dataset specifically for this purpose.

**Figure 7:**
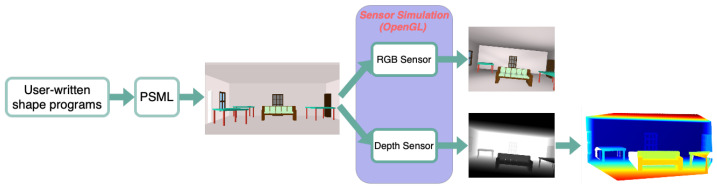
Pipeline of the different stages in the proposed data generation pipeline. The design of 3D models of scenes and objects happens in the PSML engine. These models are then passed to OpenGL, where RGB and depth sensors are simulated for rendering RGB-D images and associated ground truth labels. Point clouds can be derived from depth data using the camera’s intrinsic parameters.

This section describes a novel pipeline shown in Figure 7 to synthesize image data from user-written PSML programs. Physically realistic objects and/or scenes are first designed by shape grammar rules and created using PSML programs. These scenes are then passed to a rendering engine, for example, OpenGL to produce sensor data required by the users for their applications such as simulated RGB and/or depth image data, along with associated ground truth labels, including 2D/3D bounding boxes, semantic segmentation labels, and PSML parameters.

#### 3.4.1. Scene Data Generation

**Figure 8:**
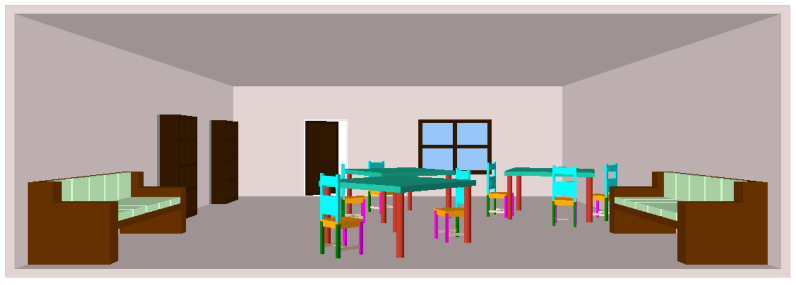
A room scene generated using PSML that combines multiple furniture objects.

Figure 8 shows a room scene consisting of different objects including tables, chairs, couches, bookshelves, a door, and a window. Shape grammar and PSML are utilized to generate 3D designs of scenes and objects within them. Users first define shape grammar programs, specifying the desired scene elements and their attributes. These programs are similar to Algorithms 1 and 2, outlining the rules governing the structure, arrangement, and characteristics of the scene components. Subsequently, the PSML engine interprets and executes these shape grammar programs, generating physically realistic 3D designs of scenes. Through this process, the PSML engine determines the spatial relationships between objects, their shapes, sizes, orientations, and other relevant properties. Users can interactively adjust the parameters and rules within the shape grammar programs to refine the generated designs according to their preferences. This integration of shape grammar and PSML allows users to efficiently generate diverse and customizable 3D designs of scenes and objects.

#### 3.4.2. Sensor Data Generation

To generate images from a scene model using a rendering engine like OpenGL, simulating camera sensors is typically necessary. By integrating a scene model with a simulated camera directed towards the scene, it becomes possible to render image data such as RGB and depth images. These rendered images can then serve as valuable input for deep learning systems, facilitating tasks such as object recognition, scene understanding, and depth estimation.

OpenGL has been previously used by other researchers as a sensor simulator [37]. In this study, both sensor values and ground truth labels are generated through OpenGL by rendering the 3D models produced by PSML into 2D images. The process of converting 3D coordinates into 2D pixels is managed by the OpenGL graphics pipeline [38], which comprises two main parts: (1) projecting 3D surface coordinates (x,y,z) to their corresponding 2D locations (x,y) in the sensor image using the sensor projection model, and (2) assigning the value of these locations to the sensed values at the projected (x,y,z) location, representing the surface appearance for RGB images and the surface-to-sensor depth for depth images.

**Figure 9:**
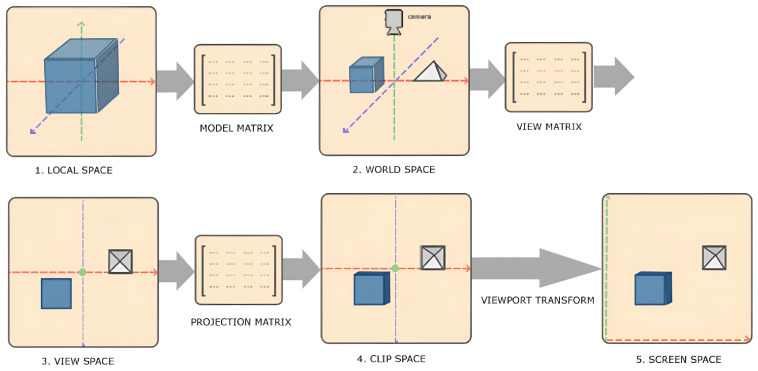
Vertex coordinate transformation from local space to screen space [38]. Object-relative vertex coordinates (local space) are converted to world coordinates (world space) using a model matrix $M_{model}$, and then world coordinates are converted to view coordinates (view space) using a view matrix $M_{view}$.

Figure 9 illustrates the OpenGL rendering pipeline and its internal transformations. In OpenGL, the transformation of local coordinates to screen (image) coordinates involves 4 steps: (1) Local-space coordinates, denoting the position of an object relative to its local origin, are transformed to world-space coordinates using a model matrix $M_{model}$. These world-space coordinates represent the object’s position relative to a broader world context and are referenced against a global origin shared by multiple objects within the scene. (2) The world coordinates are converted to view-space coordinates using a view matrix $M_{view}$, aligning them with the perspective of the camera or viewer. This transformation ensures that each coordinate reflects the object’s appearance from the viewpoint of the observer. (3) The view-space coordinates are projected to clip-space coordinates using a projection matrix $M_{projection}$, where they are processed to fit within the —1.0 and 1.0 range, determining which vertices will be visible on the screen. (4) The clip-space coordinates are transformed into screen-space coordinates through a process known as viewport transformation. The coordinates are mapped to the coordinate range defined by the viewport using the OpenGL function *glViewport*. Through this series of transformations, OpenGL accurately positions objects within the rendered scene, thereby generating the 2D OpenGL image. Steps (1–3) can be represented in the following equation, where **V** indicates the vertex and **M** indicates the transformation matrix:

(3) $$V_{clip}=M_{projection}\cdot M_{view}\cdot M_{model}\cdot V_{local}$$

The resulting screen coordinates are then forwarded to the rasterizer, where they are converted into fragments, each containing the necessary data for rendering a single pixel. The main purpose of the fragment shader is to calculate the final color of a pixel. Typically, the fragment shader contains data about the 3D scene, such as lighting, shadows, and light color, to determine the pixel’s ultimate color.

OpenGL is also employed to simulate depth sensors through the utilization of the depth buffer. The depth buffer, created by the OpenGL windowing system, stores depth values as 16-bit floats within each fragment, representing the fragment’s depth value. To mimic real depth sensors, noise consistent with actual sensors is introduced into these depth measurements as documented in the literature such as [39], which outlines observed accuracy for depth images from RGB-D sensors, like the Microsoft Kinect sensor. This depth noise follows a Gaussian model, where depth variance increases quadratically with the sensor-to-surface depth. During rendering, OpenGL compares the depth values of each fragment with the current depth buffer. Fragments that are behind other fragments are discarded, while fragments that pass this depth test are rendered, and the depth buffer is updated with the new depth values. This automated process, known as “depth testing”, is seamlessly handled by OpenGL.

#### 3.4.3. Synthetic Dataset

The capability to generate 3D models and simulate sensors offers the flexibility to generate diverse datasets tailored to specific applications. In this study, a pin-hole camera model is used as the perspective model in OpenGL to simulate the sensors for an RGB-D image dataset creation. The poses of the sensors are varied in different images. This is achieved by moving all objects in the scene in the reverse direction of camera movements, as OpenGL by itself is not aware of the concept of a camera [38]. Using the inverse camera model re-projection and the perfect depth map, it is also possible to calculate the 3D position of each surface in the scene. This integration of PSML and OpenGL for synthesizing data provides (1) RGB-D images, (2) the ground truth information of the object poses relative to the camera, (3) hierarchical decomposition of objects, and (4) parametric representation of objects, where (1), (3) and (4) benefit from PSML scene generation and (2) from the OpenGL sensor simulation.

This versatile data generation framework extends to diverse research goals, facilitating tasks such as city scene modeling with accurate labeling, object part segmentation with component-level ground truth labeling, and analysis of various object realizations to address data scarcity issues. Additionally, customization for different sensor types or views, such as fish-eye cameras or bird’s-eye view perspectives, and adjustments to illumination settings in OpenGL, further expand the framework’s applicability across varied research domains.

## 4. Results

This section presents the results of three experiments. The results demonstrate the benefits offered by the PSML shape generation method and its fusion with deep learning techniques.

### 4.1. Comparison with Other Generative Methods

This experiment was conducted to demonstrate the advantages of 3D models generated using the PSML programs over the models generated by other competing methods. Specific cases of shapes generated by different methods were analyzed. From a wide array of possible algorithms, three algorithms representing VAE, GANs, and stable diffusion, respectively, were evaluated against the PSML approach: (1) 3D Shape Variational Autoencoder (3DSVAE) [1], (2) 3D Generative Adversarial Network (3DGANs) [4], and (3) Score Jacobian Chaining (SJC) [9]. While many algorithms are available in the literature, the selected algorithms provide a representative sampling of generative methods for 3D shapes.

**Figure 10:**
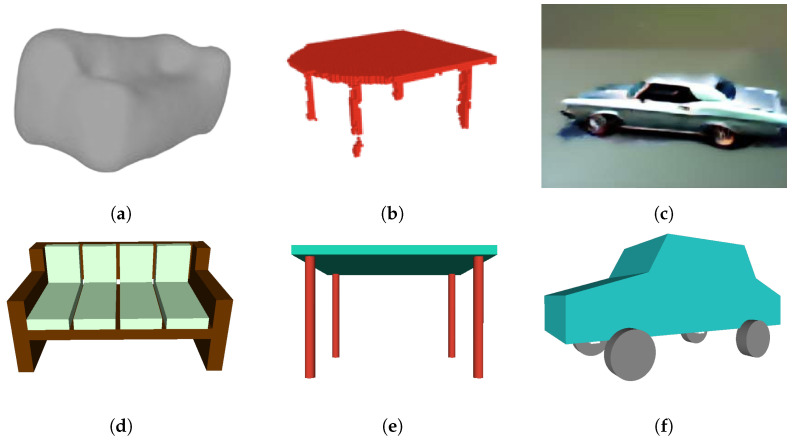
(**a**) A VAE-generated couch model [1]. (**b**) A 3D-GANs-generated table model [4]. (**c**) A stable-diffusion-generated car model [9]. (**d**–**f**) Models generated using PSML programs.

Figure 10 illustrates the comparison between 3D shapes generated using PSML and using other approaches, including VAE, GANs, and stable diffusion. The examples for comparison were sourced from their respective papers. The couch model generated by 3DSVAE (Figure 10a) lacks the structural characteristics of a couch object, offering only a rough approximation of its complex geometry. In contrast, the couch model generated using the PSML approach (Figure 10d) contains sufficient geometric features to represent a couch object. In the case of the table model generated by 3DGANs, the second leg from the left lacks manifold geometry, resulting in a discontinuous geometry and an unrealistic gap. Additionally, the legs lack self-similarity and self-symmetry in terms of size and length, which are typically present in real-world manufactured table objects. Conversely, the table model generated using PSML (Figure 10e) is a manifold polygon model and satisfies the geometric and physical constraints, attributed to its rule-based volumetric generation method. The car model generated by SJC (Figure 10c) lacks fine-grained details and fails to adhere to physical constraints, as one of the front wheels occupies the spatial location intended for the car’s front. Its PSML-generated counterpart (Figure 10f), however, presents a high-quality and physically realistic model.

Although the objects in Figure 10d–f may be rigid and visually simplistic, they do satisfy important common constraints for these commonplace objects. These constraints, for example, closed-shape geometry and free-standing capability, are necessary to be exhibited for objects to be classified into the correct category. The objects in Figure 10a–c, while being visually sophisticated, would fail most realistic tests for symmetry and usability; for example, the couch cannot be sat on, the table does not stand, and the car wheels do not roll.

The PSML approach ensures the generation of 3D models that adhere to essential geometric constraints. Expanding upon this technology to integrate more realistic details holds the potential to produce visually captivating and functionally reliable 3D geometries. This advancement could bridge the gap between visually appealing designs and practical usability, offering a holistic solution for various applications.

### 4.2. Comparison with Other Data Representations

This experiment was conducted to demonstrate the efficiency of the compact parametric shape representation offered by the PSML approach.

**Figure 11:**
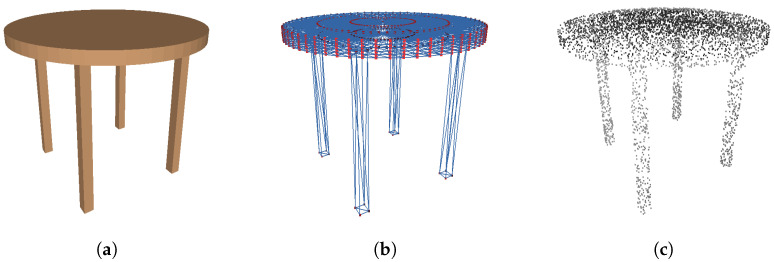
Shape grammar representation requires much less data to describe object geometry. (**a**) A table generated using Algorithm 1. Only 6 parameters are required to represent the geometry and 3 more parameters to describe the color appearance. (**b**) A polygonal mesh representation of the table with 674 vertices (blue) and 1328 points (red), each of which requires 3 parameters to represent the 3D coordinates. (**c**) A sampled point cloud from the mesh representation with 5000 3D points.

Figure 11 shows a table generated using Algorithm 1, and its polygonal mesh and point cloud representations. The shape grammar representation only requires six parameters (*l*, *w*, *h*, *t*, offset_w, and offset_l) to represent the geometry and three more parameters to describe the color appearance. In contrast, the polygonal mesh contains 674 vertices and 1328 triangular faces. The point cloud sampled from the mesh representation contains 5000 3D points. Assuming the data are represented using single-precision floating points (4 bytes), the total memory usage is 24,024 bytes for the polygonal mesh, 60,000 bytes for the point cloud representation, and only 36 bytes for the PSML parametric representation. The PSML representation requires to work with the associated program. Assuming each character in Algorithm 1 is represented using 2 bytes, the program occupies 1662 bytes, making the total memory necessitated for a PSML table object 1698 bytes. Compared to the other two representations, this parametric representation reduces the data required to describe the geometry by ∼14 times compared to the polygonal mesh and ∼35 times to the point cloud.

Table 1 illustrates the memory usage of three representations for various object instances. The memory usage for the point cloud representation is determined by sampling 2000 points per area unit of the mesh, and the PSML usage includes the memory required for the source code. The results indicate that PSML substantially reduces memory usage for most object instances, except for the chair, where polygon meshes achieved minimal usage. As the complexity of objects increases, such as in a room scene model containing various furniture pieces, the efficiency of PSML parametric representation becomes increasingly significant.

The results presented herein underscore the data efficiency offered by parametric representation in contrast to alternative methods. Through parameterization, the PSML approach retains considerable potential for achieving high data efficiency. This efficiency not only conserves memory but also streamlines the transmission and processing of object information, rendering it particularly advantageous for applications constrained by limited resources or bandwidth.

The compact parametric representation also sets the foundation for the benefits of utilizing deep learning techniques for parameter estimation, particularly in reducing the complexity of the solution space. By leveraging the compact representation, the fusion of PSML and deep learning methods promises the effective estimation of 3D shapes with greater efficiency.

### 4.3. PSML-DL Fusion for Shape Detection Task

In this experiment, a synthetic dataset is generated, and the 3DETR-P network is trained on this dataset to detect 3D objects in the scene and estimate the associated PSML parameters.

#### 4.3.1. Synthetic Dataset Generation

An experiment is conducted to demonstrate that object models can be used to synthesize training data for DL systems, improving current 3D model databases which use static 3D models and therefore lack geometric diversity.

A PSML program of the indoor room scene is written that involves six other PSML programs of common indoor furniture: table, chair, couch, bookshelf, window, and door. The proposed human-in-the-loop approach fixes various attributes of this shape-generation process and allows other aspects to vary. Fixed aspects include the size of the room and some relative and physical constraints between objects, including that (1) all of the objects are on the ground, (2) bookshelves are always against the wall, and (3) solid objects do not overlap with each other. The variations include the occurrence, location, orientation, and structural characteristics of the furniture. This is achieved by controlling the PSML parameters for each object type. These parameters are set to follow uniform distributions and to ensure realism while adhering to relative constraints within and among objects. For example, the length and width of the table object are uniformly generated from 1.5 to 2.5 units. Similarly, the height of the chair seats ranges from 0.5 to 0.8 units, reflecting real-world proportions, where chairs typically sit lower than adjacent tables.

An RGB-D image dataset of the room scene is generated using the method in Section 3.4. The dataset is then utilized in a deep learning task for detecting 3D objects within the room, where the objective is to predict the 3D bounding boxes for each object based on the input point cloud. The point cloud data are derived from the depth data using the cameras’ intrinsic parameters. Ground truth data are generated for each sample, comprising a semantic label, 3D bounding box (location, orientation, and size), and 5 PSML parameters. Specifically, for the bookshelf object, these PSML parameters include length, width, height (to define the object’s 3D dimensions), number of horizontal panels, and vertical panels (to describe its structural characteristics). While the bookshelf necessitates all five parameters for PSML generation, it is important to note that not all objects require the same number of parameters. For instance, the door object in Algorithm 2 only requires three parameters as program arguments; in such cases, the two extra parameters are set to 0. In the dataset generated, non-zero PSML parameters always precede zero parameters within the parameter vector. Among the six object classes—table, chair, couch, bookshelf, window, and door—the respective counts of non-zero PSML parameters are 4, 3, 5, 5, 4, and 3. The DL estimation of PSML parameters can be adjusted by increasing or decreasing the parameters for prediction. For example, limiting DL models to estimate only three parameters will result in less constraint within the DL solution space.

**Figure 12:**
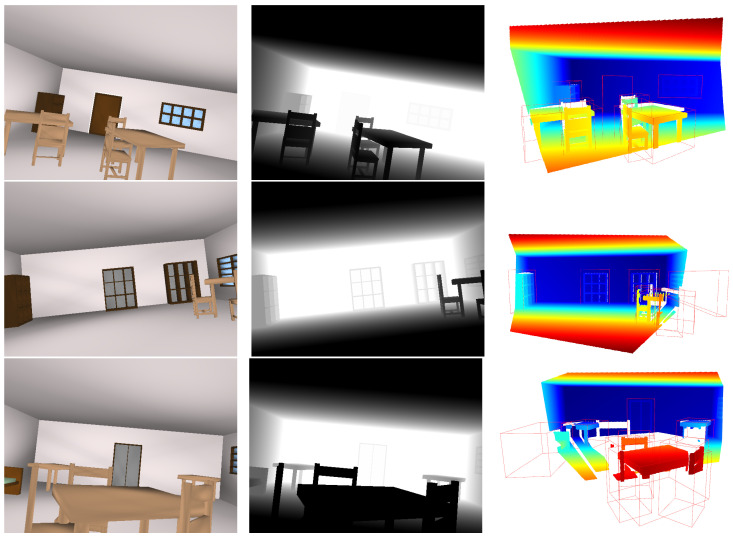
Examples in the synthetic dataset. Each row is one sample from the dataset. The images on each row from left to right are the RGB image, depth image, and point cloud, with the 3D bounding box ground truth shown in green. The point clouds are reconstructed from the depth image using the intrinsics of the simulated cameras. The orientation of the point clouds is adjusted so that the floor plane is positioned horizontally relative to the viewer’s perspective, facilitating easier interpretation and analysis of the spatial layout of the scene.

Figure 12 shows the RGB-D image pair and associated point cloud of 3 samples from the dataset containing 2000 samples. It can be seen that the occurrence of the objects, their shapes (length, width, height, etc.), positions, and orientations are different in the room space but still obey the physical constraints. Different data types serve distinct purposes across various tasks, depending on the inputs involved. For instance, point cloud data can serve as input for deep learning models engaged in tasks such as 3D object segmentation and scene completion. RGB and depth image data can be utilized either independently or collectively as inputs for deep learning models focusing on 2D image data tasks, such as 2D object segmentation and scene reconstruction from image(s). The generated synthetic dataset demonstrates the application of PSML to deep learning systems.

Throughout our experiments, the point cloud data are exclusively used as input, aligning with the architectural design of the 3DETR-P network. The point clouds are reconstructed from the depth image using the intrinsics of the simulated cameras. Both RGB and depth images are rendered at a resolution of 640 × 480. The total processing time, including rendering and file writing, for each sample ranges from 1 to 2 s on an NVIDIA GeForce RTX 4090 GPU.

#### 4.3.2. Three-Dimensional Object Detection and Shape Estimation

An experiment is conducted to demonstrate the capability of the proposed DL fusion in computer vision tasks, specifically detecting objects and estimating their parametric representation. Key metrics for the model estimates are presented, highlighting the benefits of this approach.

The original 3DETR implementation [40] is adapted and modified for the implementation of 3DETR-P using PyTorch. The standard “nn.MultiHeadAttention” module is used to implement the Transformer architecture. To process the input point cloud data, a single set aggregation operation is used to reduce the number of points to 2048 and extract 256-dimensional point features. Dropout [41] regularization for all MLPs and self-attention modules in the model, with a dropout rate of 0.1, except in the decoder, with a dropout rate of 0.3, is used to prevent overfitting. For optimization, the AdamW optimizer [42] is used with a learning rate decayed by a cosine learning rate schedule [43] to $10^{-6}$, a weight decay of 0.1, and gradient clipping at an L2 norm of 0.1. The weight for $L_{\mathrm{PSML}}$ loss in Equation (1) is set to 3. The training is performed on a NVIDIA GeForce RTX 4090 GPU for 350 epochs with a batch size of 16. Other parameters are configured to be consistent with the [36].

The dataset generated in Section 4.3.1 is split into train, validation, and test sets with 1200, 400, and 400 samples, respectively (60%–20%–20%). The 3DETR-P network designed in Section 3.3 is trained to detect 3D objects in the scene and estimate the associated PSML parameters.

**Figure 13:**
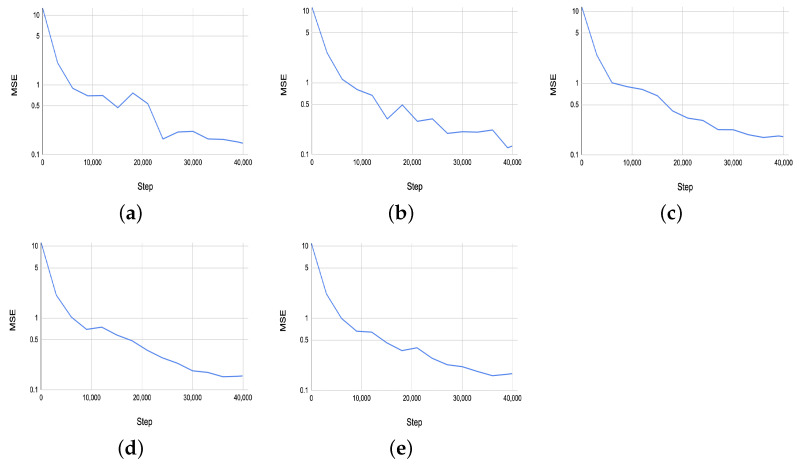
(**a**–**e**) The MSE results on the validation set for the estimated 5 PSML parameters across the training steps.

Figure 13 shows the MSE results on the validation set for the estimated five PSML parameters across training steps. The performance of the 3DETR-P network, as evaluated by the MSE on the validation set, demonstrates consistent convergence across the five estimated PSML parameters. All parameters show a steady decline in MSE throughout the training and validation processes, ultimately converging to values close to 0.1. This indicates that the 3DETR-P network is able to predict the shape parameters well, achieving a low error rate on the validation data.

Table 2 shows the testing results of the trained network. Following the practice in [36], the detection performance is reported on the test set using the mean Average Precision (mAP) at two different IoU (Intersection of Union) thresholds of 0.25 and 0.5, denoted as $AP_{25}$ and $AP_{50}$. The PSML parameters are evaluated by calculating the mean absolute error (MAE) between the estimation and ground truth. The row corresponding to 3DETR-P in the table presents its performance on the room dataset created within this study. Overall, it succeeds in detecting objects within the scene, although its performance on door detection is comparatively lower. This discrepancy may be attributed to the fact that doors in the scene often (1) lack sufficient thickness to be distinctly separated from the wall they are embedded within and (2) lack sufficient depth variations within the object to provide more features for the network to learn the structure. The $MAE_{PSML}$ row denotes the MAE of the PSML parameters, quantitatively showcasing the success of estimating the 3D shapes from the input point cloud.

Table 2 also includes the $AP_{25}$ results of naive 3DETR on other datasets, reported in [36]. The row corresponding to 3DETR-SUN reflects the 3DETR results from [36] on the SUN-RGBD dataset [44] and the 3DETR-SN row shows results on the ScanNetV2 dataset [45]. Although a direct comparison between the results in this article and theirs is not possible, it can be seen that 3DETR-P on the generated synthetic dataset achieves comparative detection performance to 3DETR on the ScanNetV2 dataset for classes like chair, couch, and door, and outperforms 3DETR for other classes. The detection performance, together with the MAE results of the estimated PSML parameters, indicates the capability of the proposed PSML and DL fused system in detecting objects and their parametric representation.

**Figure 14:**
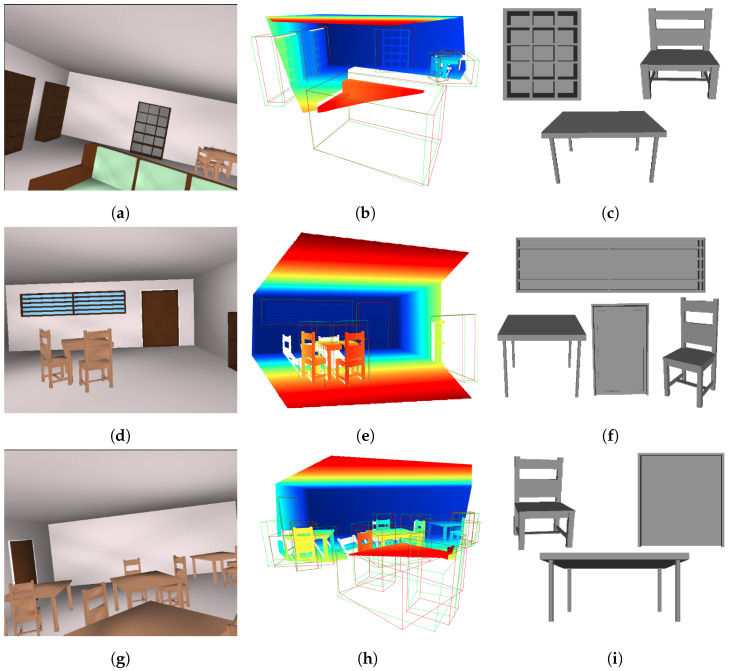
RGB image of scenes (**a**,**d**,**g**), input point cloud to the 3DETR-P with the ground truth and predicted bounding boxes in red and green, respectively (**b**,**e**,**h**), and some of the estimated 3D shapes reconstructed using the PSML parameters estimated by 3DETR-P and PSML programs (**c**,**f**,**i**).

Figure 14 visualizes three examples, presenting (1) the RGB image of the scene (left), (2) the input point cloud to 3DETR-P (middle) with the ground truth (red) and predicted 3D bounding boxes (green), and (3) the 3D shapes reconstructed using the PSML parameters estimated by 3DETR-P (right). The appearance of the reconstructed shapes is omitted, as such information is not estimated by the network in this experiment. The reconstructed 3D shapes closely resemble those observed in the RGB images and the point cloud, qualitatively demonstrating the success of 3D shape estimation from the input point cloud.

Table 3 shows the MSE results of the PSML parameter estimation (5 parameters represented by $p_{1}$–$p_{5}$) applied to the three instances depicted in Figure 14. In this analysis, the MSE values for various objects belonging to the same class are aggregated for clarity. As indicated in Section 4.3.1, among the objects categorized as table, chair, bookshelf, window, and door, the counts of non-zero parameters are 4, 3, 5, 4, and 3, respectively. It is worth noting that within the parameter vector, non-zero parameters consistently appear before zero parameters. For instance, within the table class, parameters $p_{1}$ through $p_{4}$ are non-zero, while $p_{5}$ is set to zero. The estimation errors for the zero parameters as shown in the table range from 0 to 0.02, while MSE values for the non-zero parameters range from 0.06 to 0.17. These findings further corroborate the performance reported in Table 2, demonstrating the efficacy of the 3DETR-P network in accurately estimating shape grammar parameters from 3D point data.

## 5. Conclusions

This article introduced a novel fusion approach that combines a generative formal model for 3D shapes with deep learning (DL) methods to enhance the understanding of geometric structures and component relationships within objects. The proposed method leverages shape grammar programs written in Procedural Shape Modeling Language (PSML) to encode complex object descriptions. By allowing users to write PSML programs that enforce fundamental rules and encode object attributes, this proposed fusion approach facilitates the generation of parametric representations for 3D shapes. One of the key strengths of the proposed approach is offering human-in-the-loop control over DL estimates; users can specify candidate objects, shape variations, and the level of detail, providing flexibility and control over the generated shapes. By enabling the more accurate and controllable generation of 3D shapes, this fusion of generative modeling with DL enhances the interpretability of DL estimates and offers AI models a deeper understanding of object geometry and relationships, opening up new avenues for applications in computer graphics, robotics, and virtual reality.

## Figures and Tables

**Table 1:** Memory in byte required by different representations.

	Table (Box)	Chair	Couch	Bookshelf	Window	Door	Room
PSML	1046	6410	3606	2350	2244	798	21,328
Polygon Mesh	92,496	3840	3360	15,600	18,960	1920	529,344
Point Cloud	63,360	42,000	7,063,680	647,760	786,960	192,240	14,986,080

**Table 2:** Per-class performance for 3D object detection and shape estimation. $MAE_{PSML}$ denotes the MAE of the PSML parameters. The 3DETR-P results are reported on the dataset generated in this article. The 3DETR-SUN row shows the 3DETR results from [36] on the SUN-RGBD dataset, where “-” indicates that such measurement is not available. The 3DETR-SN row shows the results of 3DETR on the ScanNetV2 dataset.

		Table	Chair	Couch	Bookshelf	Window	Door	Overall
3DETR-P	$AP_{25}$	98.90	87.60	99.30	98.95	93.89	56.84	89.25
	$AP_{50}$	89.64	61.76	95.16	93.96	62.51	13.16	69.36
	$MAE_{PSML}$	0.14	0.11	0.19	0.16	0.23	0.20	0.17
3DETR-SUN	$AP_{25}$	52.6	72.4	65.3	28.5	-	-	54.7
3DETR-SN	$AP_{25}$	67.6	90.9	89.8	56.4	39.6	52.4	66.1

**Table 3:** The MSE results from estimating the PSML parameters for the three examples depicted in Figure 14. In this context, $p_{1}$–$p_{5}$ represent the five estimated parameters, with the MSE values averaged across different objects within the same class.

		$p_{1}$	$p_{2}$	$p_{3}$	$p_{4}$	$p_{5}$
Exp 1 (Figure 14c)	table	0.13	0.06	0.12	0.12	0
	chair	0.10	0.10	0.15	0	0.01
	bookshelf	0.08	0.13	0.07	0.11	0.14
Exp 2 (Figure 14f)	table	0.09	0.11	0.08	0.12	0.01
	chair	0.07	0.09	0.13	0	0
	window	0.17	0.13	0.17	0.15	0
	door	0.14	0.16	0.13	0.01	0.01
Exp 3 (Figure 14i)	table	0.12	0.10	0.09	0.11	0
	chair	0.12	0.09	0.13	0.01	0
	door	0.13	0.15	0.12	0	0.02

## Data Availability

The raw data supporting the conclusions of this article will be made available by the authors on request.

## Abbreviations

    The following abbreviations are used in this manuscript:

3D	Three-dimensional
DL	Deep Learning
PSML	Procedural Shape Modeling Language
AI	Artificial Intelligence
VAE	Variational Autoencoders
GANs	Generative Adversarial Networks
MAE	Mean Absolute Error
3DSVAE	3D Shape Variational Autoencoder
3DGANs	3D Generative Adversarial Network
SJC	Score Jacobian Chaining
